# Supplementary material for: Examining doctors’ business analytics capabilities in using the electronic medical record system for decision-making effectiveness in intensive care units: Impact of the COVID-19 pandemic
Source: PLoS One. 2025 Jul 1;20(7):e0317954. doi: 10.1371/journal.pone.0317954 (PMC12212584; doi:10.1371/journal.pone.0317954)
Supplement: S1 Table — (DOCX) [file pone.0317954.s001.docx]

**S1 Table. Appendix: Survey Instruments**

| **Construct** | **Item** | **Description** |
| --- | --- | --- |
| Please indicate how much you agree or disagree to the following: | | |
| Perceived Usefulness  *Adapted from* [Sykes, Venkatesh [39]](#_ENREF_39) | PU01 | I believe EMR would be useful in my job. |
|  | PU02 | Using EMR will enable me to accomplish tasks more quickly. |
|  | PU03 | Using EMR will increase my productivity. |
|  | PU04 | If I use EMR, I will increase my chances of getting a raise. |
| Perceived Ease of Use  *Adapted from* [Sykes, Venkatesh [39]](#_ENREF_39) | PEU01 | My interaction with EMR would be clear and understandable. |
|  | PEU02 | It would be easy for me to become skillful at using EMR. |
|  | PEU03 | I would find EMR to be easy to use. |
|  | PEU04 | Learning to operate EMR would be easy for me. |
| Please indicate how much you agree or disagree that EMR can effectively enable you to do the following: | | |
| Data aggregation  *Adapted from* [Wang and Byrd [6]](#_ENREF_6) | DAG01 | Collect data from various health systems throughout our hospital and external healthcare sources. |
|  | DAG02 | Make patient records consistent, visible and easily accessible for further analysis. |
|  | DAG03 | Store patient data into appropriate databases. |
| Data analysis  *Adapted from* [Wang and Byrd [6]](#_ENREF_6) | DAN01 | Identify important business insights and trends to improve healthcare services. |
|  | DAN02 | Predict patterns of care in response to patient needs. |
|  | DAN03 | Analyze data in near-real or real time that allows responses to unexpected clinical events. |
|  | DAN04 | Analyze social media data to understand current trends from a large population. |
| Data interpretation  *Adapted from* [Wang and Byrd [6]](#_ENREF_6) | DIT01 | Provide systemic and comprehensive reporting to help recognize feasible opportunities for care improvement. |
|  | DIT02 | Support data visualization that enables us to easily interpret results. |
|  | DIT03 | Provide near-real or real-time information on health care operations and services within healthcare facilities and across health care systems. |
| Decision-making effectiveness  *Adapted from* [Wang and Byrd [6]](#_ENREF_6) | DME01 | As a result of EMR, the quality of decisions has improved. |
|  | DME02 | As a result of EMR, the speed at which decisions are analyzed has increased. |
|  | DME03 | As a result of EMR, our hospital staffs have an increased understanding of our patients. |

Note: All questions were asked using a 7-point Likert’s scale.
