# Supplementary material for: Examining doctors’ business analytics capabilities in using the electronic medical record system for decision-making effectiveness in intensive care units: Impact of the COVID-19 pandemic
Source: PLoS One. 2025 Jul 1;20(7):e0317954. doi: 10.1371/journal.pone.0317954 (PMC12212584; doi:10.1371/journal.pone.0317954)
Supplement: S2 Table — (DOCX) [file pone.0317954.s002.docx]

**S2 Table. Appendix: Common Method Bias Assessment**

Based on a latent common method factor approach ([50](#_ENREF_50)) using PLS-SEM ([51](#_ENREF_51)), a method factor was added to the structural model to load all items to the respective latent constructs and the latent method factor. Specifically, each item was converted to a single-item first-order construct, whereas all the latent constructs and the method factor became second-order constructs. This conversion ensured that each single-item construct was statistically equivalent to a single observed indicator in the structural model with no information loss ([51](#_ENREF_51), [52](#_ENREF_52)). Consequently, the variance of each item was partitioned into its substantive construct, the method factor, and random measurement error. The common method bias assessment shows that all factor loadings of the method factor were not significant at the 1% significance level. Furthermore, the method factor explained only 0.8% average variance in the data, while the average variance of the substantive constructs was 83.3%, yielding a 1:102 ratio of method variance to substantive variance. We can conclude insignificant and small magnitudes of method variance. Hence, common method bias was unlikely a severe concern for this study.

|  |  | **Substantive Factor Loading (R1)** | **R1^2^** | **Method  Factor Loading (R2)** | **R2^2^** |
| --- | --- | --- | --- | --- | --- |
| Data aggregation | DAG1 | 0.814*** | 0.663 | 0.099 | 0.010 |
|  | DAG2 | 0.954*** | 0.910 | 0.002 | 0.000 |
|  | DAG3 | 1.035*** | 1.071 | -0.097* | 0.009 |
| Data analysis | DAN1 | 0.934*** | 0.872 | 0.001 | 0.000 |
|  | DAN2 | 0.862*** | 0.743 | 0.055 | 0.003 |
|  | DAN3 | 0.798*** | 0.637 | 0.108 | 0.012 |
|  | DAN4 | 1.021*** | 1.042 | -0.173 | 0.030 |
| Data interpretation | DIT1 | 0.929*** | 0.863 | 0.010 | 0.000 |
|  | DIT2 | 0.932*** | 0.869 | 0.007 | 0.000 |
|  | DIT3 | 0.948*** | 0.899 | -0.018 | 0.000 |
| Decision-making effectiveness | DME1 | 0.982*** | 0.964 | -0.070 | 0.005 |
|  | DME2 | 0.958*** | 0.918 | -0.004 | 0.000 |
|  | DME3 | 0.862*** | 0.743 | 0.074 | 0.005 |
| Perceived ease of use | PEU1 | 0.853*** | 0.728 | -0.017 | 0.000 |
|  | PEU2 | 0.902*** | 0.814 | 0.021 | 0.000 |
|  | PEU3 | 0.844*** | 0.712 | 0.087* | 0.008 |
|  | PEU4 | 0.987*** | 0.974 | -0.095 | 0.009 |
| Perceived usefulness | PU1 | 0.976*** | 0.953 | -0.099 | 0.010 |
|  | PU2 | 0.987*** | 0.974 | -0.058 | 0.003 |
|  | PU3 | 0.975*** | 0.951 | -0.033 | 0.001 |
|  | PU4 | 0.432** | 0.187 | 0.254 | 0.065 |
| **Average** |  | **0.904** | **0.833** | **0.003** | **0.008** |

*** p < 0.001, ** p < 0.01, * p < 0.05
