# Supplementary material for: Examining doctors’ business analytics capabilities in using the electronic medical record system for decision-making effectiveness in intensive care units: Impact of the COVID-19 pandemic
Source: PLoS One. 2025 Jul 1;20(7):e0317954. doi: 10.1371/journal.pone.0317954 (PMC12212584; doi:10.1371/journal.pone.0317954)
Supplement: S3 Table — (DOCX) [file pone.0317954.s003.docx]

**S3 Table. Appendix: Structural Model Results based on Pooled Sample**

| Hypothesized Paths | | Direct Effects | Total Indirect Effects | Total Effects |
| --- | --- | --- | --- | --- |
| H1a | PEU 🡪 DAG | 0.214 (0.133) | 0.303 (0.015) ** | 0.517 (0.000) *** |
| H1b | PEU 🡪 DAN | 0.202 (0.145) | 0.297 (0.017) ** | 0.499 (0.000) *** |
| H1c | PEU 🡪 DIT | 0.205 (0.137) | 0.264 (0.049) ** | 0.468 (0.000) *** |
|  | PEU 🡪 DME |  | 0.398 (0.000) *** | 0.398 (0.000) *** |
| H2a | PU 🡪 DAG | 0.359 (0.013) ** |  | 0.359 (0.013) ** |
| H2b | PU 🡪 DAN | 0.352 (0.016) ** |  | 0.352 (0.016) ** |
| H2c | PU 🡪 DIT | 0.313 (0.049) ** |  | 0.313 (0.049) ** |
|  | PU 🡪 DME |  | 0.272 (0.021) ** | 0.272 (0.021) ** |
| H3 | PEU 🡪 PU | 0.843 (0.000) *** |  | 0.843 (0.000) *** |
| H4a | DAG 🡪 DME | 0.225 (0.042) ** |  | 0.225 (0.042) ** |
| H4b | DAN 🡪 DME | 0.188 (0.275) |  | 0.188 (0.275) |
| H4c | DIT 🡪 DME | 0.400 (0.006) *** |  | 0.400 (0.006) *** |
| Note: Significance level: *** p < .01; ** p <.05; * p <.10. DAG: Data Aggregation; DAN: Data Analysis; DIT: Data Interpretation; PEU: Perceived Ease of Use; PU: Perceived Usefulness; DME: Decision-Making Effectiveness. | | | | |
