# Supplementary material for: Examining doctors’ business analytics capabilities in using the electronic medical record system for decision-making effectiveness in intensive care units: Impact of the COVID-19 pandemic
Source: PLoS One. 2025 Jul 1;20(7):e0317954. doi: 10.1371/journal.pone.0317954 (PMC12212584; doi:10.1371/journal.pone.0317954)
Supplement: S4 Table — (DOCX) [file pone.0317954.s004.docx]

**S4 Table. Appendix: Inter-construct Correlation Matrix**

Collinearity in the structural model was evaluated based on inner VIF following Hair et al.’s [47] guideline, even though the need of reporting it was not indicated in Benitez et al. (2020). Benitez et al. [49] suggested reporting inter-construct correlations to indicate the strength of relationship between constructs. Table S4-1 and S4-2 show the construct correlation matrix for Time 1 and Time 2, respectively.

Table S4-1: Construct Correlation Matrix (Time 1)

| **Constructs** | **DAG** | **DAN** | **DIT** | **DME** | **PEU** | **PU** |
| --- | --- | --- | --- | --- | --- | --- |
| **DAG** | 1.000 |  |  |  |  |  |
| **DAN** | 0.788 | 1.000 |  |  |  |  |
| **DIT** | 0.641 | 0.784 | 1.000 |  |  |  |
| **DME** | 0.689 | 0.769 | 0.840 | 1.000 |  |  |
| **PEU** | 0.672 | 0.642 | 0.594 | 0.649 | 1.000 |  |
| **PU** | 0.687 | 0.607 | 0.539 | 0.642 | 0.847 | 1.000 |

Note: DAG: Data Aggregation; DAN: Data Analysis; DIT: Data Interpretation; DME: Decision-Making Effectiveness; PEU: Perceived Ease of Use; PU- Perceived Usefulness

Table S4-2: Construct Correlation Matrix (Time 2)

| **Constructs** | **DAG** | **DAN** | **DIT** | **DME** | **PEU** | **PU** |
| --- | --- | --- | --- | --- | --- | --- |
| **DAG** | 1.000 |  |  |  |  |  |
| **DAN** | 0.863 | 1.000 |  |  |  |  |
| **DIT** | 0.854 | 0.945 | 1.000 |  |  |  |
| **DME** | 0.690 | 0.725 | 0.667 | 1.000 |  |  |
| **PEU** | 0.206 | 0.244 | 0.237 | 0.182 | 1.000 |  |
| **PU** | 0.244 | 0.339 | 0.340 | 0.298 | 0.815 | 1.000 |

Note: DAG: Data Aggregation; DAN: Data Analysis; DIT: Data Interpretation; DME: Decision-Making Effectiveness; PEU: Perceived Ease of Use; PU- Perceived Usefulness
