# Supplementary material for: Examining doctors’ business analytics capabilities in using the electronic medical record system for decision-making effectiveness in intensive care units: Impact of the COVID-19 pandemic
Source: PLoS One. 2025 Jul 1;20(7):e0317954. doi: 10.1371/journal.pone.0317954 (PMC12212584; doi:10.1371/journal.pone.0317954)
Supplement: Attachment — Submitted filename: Response to Reviewers.docx [file pone.0317954.s006.docx]

**PONE-D-22-18704**

**Examining doctors’ business analytics capabilities in using the electronic medical record system for decision-making effectiveness in intensive care units: impact of the COVID-19 pandemic**

**Authors’ responses to reviewers’ comments:**

Dear editors and reviewers,

First of all, thank you for providing us with the opportunity to submit a revised version of our manuscript, *“Examining doctors’ business analytics capabilities in using the electronic medical record system for decision-making effectiveness in intensive care units: impact of the COVID-19 pandemic”,* for publication consideration in PLOS ONE.

We thank all reviewers for their valuable comments. The positive feedback and compliments have certainly encouraged us to strive for excellence in our research. We very much appreciate the encouraging and useful comments given in response to our manuscript submission. Our revision takes *all* the suggested areas of improvement provided by the reviewers into account to improve our manuscript further. Below is a summary of our revision. Our detailed responses to each of the reviewers’ comments can be found on the following pages.

Please also find additional information requested by the academic editor in our cover letter. All relevant data are within the paper and available on the *figshare* public data repository. Please find the anonymous, private reviewer URL to our minimal data set here: <https://figshare.com/s/d9cd0f8d81a4c284f919>

We are confident that you will find we have been able to address all of the reviewers’ comments satisfactorily and hope that you will find the revised manuscript suitable for publication in your esteemed journal. Thank you again for inviting us to re-submit our manuscript.

Best regards,

*Anonymous authors*

| **Reviewer 1 Feedback** |  |
| --- | --- |
| The abstract needs to be revised considering the context, challenge, aim, method, results and implications. | We thank you for the positive feedback and compliments that have certainly encouraged us to strive for excellence in our research. We very much appreciate your encouraging and useful comments given in response to our manuscript submission. Our revision considers all your suggested areas of improvement to improve our manuscript further.  Thank you for your comment. We have revised the abstract thoroughly as suggested. In particular, we incorporated and/or enhanced the study’s challenge (lines 3-4), aim (lines 4-6), research questions (lines 7-9), context (lines 10-12), methods (lines 12-14), results (lines 14-19), and implications (lines 19-24) in the abstract. |
| Research questions must be included. | We have included the research questions in the abstract (p.1 lines 7-9). We have also indented and italicized the research questions for better clarity in the introduction section (p.3 lines 57-60). |
| More discussion is highly recommended for related works, research gaps, contributions and novelty.  More discussion is required for the results. | Thank you for your recommendation. We have thoroughly extended the discussion by linking to related works, research gaps, contributions, and novelty. Please refer to the revised Discussion section on pp.22-29 in the Manuscript. |
| Research limitations and potential future directions need to be added. | We renamed the conclusions section to “Conclusions, limitations, and future research” to better reflect the content in this section. We further clarified the research limitations and potential future directions related to our study (pp.30-31, lines 568-580). |
| More details must be clarified in the conclusion. | We added a key message summarizing the study’s conclusion (pp.29-30, lines 543-548):  *When doctors find the EMR system easy to use, they consequently believe that using the EMR system is useful for developing their data-related capabilities. Through the pandemic, doctors perceive less the ease of using the EMR system but emphasize its usefulness for developing their data analysis and interpretation capabilities. More specifically, doctors’ data analysis capability becomes the sole driver for making effective decisions in ICU during the pandemic.*  We further clarified the research limitations and potential future directions related to our study (pp.30-31, lines 568-580). |

| **Reviewer 2 Feedback** |  |
| --- | --- |
| The use of an electronic medical record system in developing doctors' business analytics capabilities for more effective healthcare decisions in intensive care units prior to and during the COVID-19 pandemic is investigated in this manuscript. It is critical to address doctors' opinions of the EMR system's technology in terms of its ease of use and usefulness in connection to business analytics capabilities such as data aggregation, data analysis, and data interpretation. The authors analyze and evaluate the study questions thoroughly. They also highlight good theoretical and practical consequences. | We thank you for the positive feedback and compliments that have certainly encouraged us to strive for excellence in our research. We very much appreciate the reviewer’s appreciation of our work. Our revision considers all the suggested areas of improvement provided by the reviewers to improve our manuscript further. |

| **Reviewer 3 Feedback** |  |
| --- | --- |
| This paper focuses on Doctors' business analytics capabilities in the use of the EMR system for decision making pre- and post-COVID-19. This is relevant in shaping good clinical practice. There are a few comments that need to be addressed:  1. The presentation creates the impression that this is a review paper. Since this is a Research paper, the literature review section is not needed. The literature provided should rather be used in the discussion section, which largely is not related to previous or other studies in its current format. | We thank you for the positive feedback and compliments that have certainly encouraged us to strive for excellence in our research. We very much appreciate your encouraging and useful comments regarding our work. Our revision considers all your suggested areas of improvement to improve our manuscript further.  We have removed the literature review as suggested and extended the discussion section by carefully relating to previous studies (pp.22-29 in the Manuscript).  We are confident that the revised manuscript now creates a more appropriate impression as a Research paper. |
| 2. The Research model and hypothesis should be summarized. The copious hypothesis explanation is not necessary.  - Consider referencing figure 1 after healthcare setting (i.e. ".......in the health care setting (Figure 1)")  - The line from "The Research model is illustrated..." till the end of the section can be summarized as follows:  "We analyzed four (4) hypotheses:  1. PEU has a direct positive effect on using the EMR system for DAG, DAN, and DIT  2. PU ..................................................................  3. PEU..............................................................  4a. Using the EMR system for DAG has a direct positive effect on DME  4b. Using the EMR system for DAN .........................................  4c. Using the EMR system for DIT ......................................." | We have summarized as suggested.  We referenced (Fig 1) after “the healthcare setting” and removed the following line (p.5 in the Manuscript).  We added “we analysed four (4) main hypotheses” following Fig 1 (p.5 lines 106-117).  *H1: Perceived ease of use (PEU) has a direct positive effect on using the EMR system for data aggregation (DAG; H1a), data analysis (DAN; H1b), and data interpretation (DIT; H1c).*  *H2: Perceived usefulness (PU) has a direct positive effect on using the EMR system for data aggregation (DAG; H2a), data analysis (DAN; H2b), and data interpretation (DIT; H2c).*  *H3: Perceived ease of use (PEU) has a direct positive effect on the perceived usefulness (PU) of using the EMR system.*  *H4: Using the EMR system for data aggregation (DAG; H4a), data analysis (DAN; H4b), and data interpretation (DIT; H4c) has a direct positive effect on decision-making effectiveness (DME).*  While we now have four main hypotheses (H1-H4), we also retained the notations for sub-hypotheses (H1a, H1b,…etc) in Fig 1 and the Manuscript. The inclusion would ensure clarity for readers when cross-checking between Fig 1 and the hypothesized paths tested in the structural model (especially Table 7, pp.18-19) and reported under the structural model assessment section (pp. 17-21). The notations for sub-hypotheses would also help readers to follow through the discussion section (pp. 22-29) as we discussed the interplay between the sub-dimensions of technology perceptions (PEU, PU) and business analytics capabilities (DAG, DAN, DIT) on influencing decision-making effectiveness (DME). We hope the reviewer will find our explanation satisfactory to retain the notation for sub-hypotheses in the Manuscript. |
| 3. Methods:  - The sentence from "Comparing both periods......." till the end and Table 1 are best suited for the Results section. | Thank you for pointing this out. We have revised as suggested. Table 1 and its corresponding text have been moved from the Methods section to the Results section (pp.9-11, lines 215-229). |
| 4. Discussion  - As mentioned earlier, this section is largely not related to other or previous studies. Need revision. | Thank you for your comment. We have removed the literature review as suggested and extended the discussion section by carefully relating to previous studies. Please refer to the revised Discussion section on pp.22-29 in the Manuscript. |
| 5. Conclusions:  - Authors should summarize the conclusions into a key message related to Doctors' business analytics capabilities in using EMR system for decision making pre- and post-COVID-19. | Thank you for your useful suggestion. We added a key message summarizing the study’s conclusion (pp.29-30, lines 543-548):  *When doctors find the EMR system easy to use, they consequently believe that using the EMR system is useful for developing their data-related capabilities. Through the pandemic, doctors perceive less the ease of using the EMR system but emphasize its usefulness for developing their data analysis and interpretation capabilities. More specifically, doctors’ data analysis capability becomes the sole driver for making effective decisions in ICU during the pandemic.* |
| 6. Figure 1:  - In view of comment 2 above, there is no need for keeping H1a - H4c in the figure. The direction of the arrows is self-explanatory. | Thank you for your suggestion. We respectfully decided not to revise Fig 1.  Although we have listed the four main hypotheses as H1-H4 following Fig 1 (p.5 lines 106-117), we decided to retain the notations for sub-hypotheses (H1a, H1b,…etc) in Fig 1 and the Manuscript.  While the arrow direction in Fig 1 is self-explanatory, keeping the notation for sub-hypotheses (H1a – H4c) would ensure clarity for readers when cross-checking between Fig 1 and the hypothesized paths tested in the structural model (especially Table 7, pp.18-19) and reported under the structural model assessment section (pp. 17-21).  The notations for sub-hypotheses would also help readers to follow through the discussion section (pp.22-29) as we discussed the interplay between the sub-dimensions of technology perceptions (PEU, PU) and business analytics capabilities (DAG, DAN, DIT) on influencing decision-making effectiveness (DME). We hope the reviewer will find our explanation satisfactory to retain the notation for sub-hypotheses in the Manuscript. |

--- End of Authors’ responses to reviewers’ comment ---
