# Supplementary material for: Examining doctors’ business analytics capabilities in using the electronic medical record system for decision-making effectiveness in intensive care units: Impact of the COVID-19 pandemic
Source: PLoS One. 2025 Jul 1;20(7):e0317954. doi: 10.1371/journal.pone.0317954 (PMC12212584; doi:10.1371/journal.pone.0317954)
Supplement: Attachment — Submitted filename: Response to Reviewers R2.docx [file pone.0317954.s007.docx]

**PONE-D-22-18704**

**Examining doctors’ business analytics capabilities in using the electronic medical record system for decision-making effectiveness in intensive care units: impact of the COVID-19 pandemic**

**Authors’ responses to reviewers’ comments:**

Dear editors and reviewers,

First of all, thank you for providing us with the opportunity to submit a second revised version of our manuscript, *“Examining doctors’ business analytics capabilities in using the electronic medical record system for decision-making effectiveness in intensive care units: impact of the COVID-19 pandemic”,* for publication consideration in PLOS ONE.

We thank all reviewers for their valuable comments. The positive feedback and compliments have certainly encouraged us to strive for excellence in our research. We very much appreciate the encouraging and useful comments regarding our manuscript submission. Our revision takes *all* the suggested areas of improvement the reviewers provided to improve our manuscript further. Below is a summary of our revision. Our detailed responses to each of the reviewers’ comments can be found on the following pages.

In this second revision, thanks to the 4^th^ reviewer, we enhanced the readability of our paper significantly, making it suitable for medical readers and the general audience. We have copyedited this second revised manuscript to (i) consistently use active voice where possible, except when reporting methods and results, (ii) simplify complex passive sentences with coherent and concise wordings, and (iii) re-structure/re-organize some parts of the manuscript to be more familiar to medical readers. We trust this copyedited manuscript now reads well, is coherent, and is easier to follow.

Please be assured that we have also carefully preserved the improvements we have previously made in our first revised manuscript. The additional information requested by the academic editor remains as communicated in our cover letter. All relevant data are included within the paper and available in the *figshare* public data repository. Please find the anonymous, private reviewer URL to our minimal data set here: <https://figshare.com/s/d9cd0f8d81a4c284f919>

We are confident that you will find we have been able to address all of the reviewers’ comments satisfactorily and hope that you will find the second revised manuscript suitable for publication in your esteemed journal. Thank you again for inviting us to re-submit our manuscript.

Best regards,

*Anonymous authors*

| **Reviewer 3 Feedback** |  |
| --- | --- |
| All comments have been addressed. No further response to the review questions #2 to #6. | We thank you for the assurance and the compliments that we have addressed all the comments in your previous review, and no further revision is required. |

| **Reviewer 4 Feedback** |  |
| --- | --- |
| Thank you for the opportunity to review this manuscript. The topic is interesting and important to clinicians and the study itself seems to have been rigorously done. However, if the target audience includes clinicians, I worry that in its current format, the manuscript is difficult to follow and excessively lengthy.  As a clinician and researcher, I found this paper somewhat difficult to follow, overly wordy in many places with words and phrases. I do acknowledge that the edits in response to the previous reviewers have improved the manuscript. I think the manuscript would benefit from copy editing. I will try to provide some specific suggestions but cannot address all of the statements/ sections that need more brevity. | Thank you for your comments. We are encouraged by your positive feedback that our topic is interesting and important to clinicians like your good self. We very much appreciate your useful suggestions as a clinician and researcher. Our revision considers all your suggested areas of improvement to improve our manuscript further.  We have copyedited our manuscript to enhance its structure and readability, making it suitable for medical readers and the general audience. You may find more specific changes we have made to this revised manuscript in our following responses to your comments.  Please be assured that while copyediting this second revised manuscript, we have also carefully preserved the improvements we made in our first revised manuscript. |
| General Comments - As a starting point, perhaps the authors can consistently use active voice rather than complex passive sentences. - Also, the structure of the sections are not standard for medical literature. It's more akin to a graduate thesis. Can the authors re-evaluate and re-structure the manuscript to be more familiar to medical readers?  - Would suggest using more simple statements without added adverbs. For example, the discussion starts with “the empirical results evidence…” These words are all saying the same thing. How about simply, “we found that…” Another example is in the Discussion (1st para): "... significant predictors of doctors' behavioral intension to use the system..." What are behavioral intentions? There are numerous other examples of this issue and I hope the authors can review the manuscript and address these. | We have copyedited this second revised manuscript to (i) consistently use active voice and first-person point-of-view (“we…”) where possible, except when reporting methods and results, (ii) simplify complex passive sentences with coherent and concise wordings, and (iii) re-structure/re-organize some parts of the manuscript to be more familiar to medical readers. We carefully reviewed and revised the manuscript to ensure consistent usage of words, format, and structure to enhance readability.  We also removed unnecessary words that might cause confusion to our readers. For example, we refrained from using ‘behavioral intention’, an outcome variable widely found in the information system literature but not an outcome our study measures.  We trust this copyedited manuscript now reads well, is coherent, and is easier to follow. |
| - The abstract is not structured with subheadings. Is this not a requirement by the journal? | Thank you for your comment. We have revised the abstract according to your suggestions.  Previously, our first revised abstract was written following a standard structure outlining the study’s challenge, aim, research questions, context, methods, results, and implications.  Now, we added the subheadings to guide readers through our abstract.  We referred to samples of PLoS ONE published articles for the abstract and manuscript format, e.g., Vaithilingam et al. (2023) at https://doi.org/10.1371/journal.pone.0282520 |
| Introduction - If the authors' objective was to evaluate the perceptions of doctors on the usability… etc of this EMR, could the authors consider a more succinct Introduction that simply ends with this objective statement? - The final sections of the Introduction describes study methodology. Can the authors just include this in the methods section so the Intro ends with study objectives? - Business analytics capabilities is a prominent feature of the study but is not adequately defined. Please clearly define this in the Introduction and indicate its relationship with EMR usage. This is currently highly unclear. To provide context for readers, are there any examples of the relevance of this in critical care or even in clinical medicine in general? | Thank you for your suggestions. We have restructured and reworked the Introduction to enhance the study’s focus and relevance to medical readers. In particular, we restructured the logical flow of the Introduction to align with our study’s objective—evaluating doctors’ perception of using the EMR system to develop business analytics (BA) capabilities for effective decision-making.  We ended the Introduction with a strong objective statement and two research questions (recommended by the previous reviewers). And we moved the final sections of the Introduction to more relevant sections of the manuscript i.e., the Conceptual model and the Methods sections.  The Introduction has an improved structure, briefly described as follows.  We started our introduction by emphasizing the advancement and purpose of using an EMR system in hospitals as a vital decision support tool. We incorporated the context of public tertiary hospitals that serve non-profit-driven business objectives to define and clarify decision-making effectiveness (DME) as the outcome variable of our study.  Then, we lent support from existing information systems (IS), health IS, and business analytics (BA) literature to understand why the use of EMR systems can relate with BA capabilities. We logically unfolded why we should examine the BA capabilities development from the end users’ perspectives, i.e., individual doctors behind using an EMR system.  Finally, we emphasized our study’s objective to address the arising challenge/problem we hoped to examine---when doctors are ambiguous about the benefits of using the EMR system and lack the BA-related skill sets to derive these benefits from using the EMR system for healthcare decisions. |
| Methods - Please describe the difference between specialists, medical officers, and house officers as these terms are used in some countries but not others. - Please describe what common methods bias is, if this article is intended for a medical audience, this is likely not familiar to them. | Thank you for your suggestions. W  After evaluating our study’s objective, we decided to remove this potentially confusing clause of (specialists, medical officers, and house officers) when describing our data collection process. Instead we clarified by adding the following text (2^nd^ paragraph under data collection):  *We employed the cluster random sampling method to survey ICU doctors currently attending to critical care in a public tertiary hospital and using the EMR system for decision-making. Doctors refer to individuals with MD degrees and are the primary decision-makers about patient care and diagnosis (Skyes & Venkatesh, 2011).*  We simplified the statistical analysis subheading in two ways. Firstly, the procedures for performing the PLS-SEM analysis are integrated directly into the results in the Result section where appropriate. Secondly, the procedures for assessing common method bias are moved to the supporting information in the S2 Table. We also added a line to describe what and why the common method bias assessment is necessary to ensure robustness in our analysis. |
| Results - The results section dives right into the main findings without describing the sample, response rates, etc. Please describe the study sample first (currently somewhat described in second paragraph). - What were the response rates? (ie, how many were invited and how many responded partially and fully?) | Thank you for pointing out a potential improvement to our manuscript.  We revised the first two paragraphs into a single paragraph under the Results section to present summary statistics of the final sample consisting of 130 valid responses.  For clarification, we have previously included the samples and sampling procedure under the Methods > Data collection and sampling design. With the revision of the first paragraph in the Result, we mitigate the risk of readers missing the study’s sample description.  As described in the manuscript (2^nd^ paragraph under data collection), the survey invitation was sent out to all doctors involved in critical care at the selected ICUs. Time 1 survey was distributed using paper-based and online questionnaires before the pandemic. The time 2 survey was administered using online questionnaires only during the pandemic due to access limitations.  While the study aimed to administer a questionnaire survey to all doctors working in the selected cluster, namely the ICUs, we could only reach out to doctors involved in the immediate care of critically ill patients in the ICU who were present during our visit to the hospital. Our sampling frame was also limited to doctors in ICUs equipped with an EMR system in the Klang Valley region of Malaysia. Regrettably, we do not have the base value to compute our response rate because the full list of ICU doctors on duty was unavailable, especially due to the high staff movement during the pandemic.  As such, we clarify the responses by adding the following text (2^nd^ paragraph under data collection): We could only recruit ICU doctors who were present during our visit to the hospital before the pandemic (Time 1) and through snowballing contacts among the closely knitted ICU doctor groups in the hospital during the pandemic (Time 2). |
| Discussion - The Discussion covers the literature well, but is overly lengthy as well. - Is there a need for the Discussion to include a page on "Theoretical implications and contributions" and "Practical implications and contributions"? | Thank you for your recommendation. The Discussion section was thoroughly extended in our first revision, linking it with related works, research gaps, contributions, and novelty as suggested by previous reviewers. Unwittingly, this section became overly lengthy.  In this second revision, we put considerable effort into re-evaluating and restructuring the Discussion section. Where appropriate, we moved some discussions to the Introduction and the Conceptual model sections so that readers could follow the study’s research gaps and novelty early in the manuscript as we unfolded the examination. We trimmed down unnecessary words and section subheadings that might cause confusion to readers. We also simplified sentences to craft a coherent and logical Discussion section. |

--- End of Authors’ responses to reviewers’ comment ---
