# Supplementary material for: Examining doctors’ business analytics capabilities in using the electronic medical record system for decision-making effectiveness in intensive care units: Impact of the COVID-19 pandemic
Source: PLoS One. 2025 Jul 1;20(7):e0317954. doi: 10.1371/journal.pone.0317954 (PMC12212584; doi:10.1371/journal.pone.0317954)
Supplement: Attachment — Submitted filename: Response to Reviewers R3.docx [file pone.0317954.s008.docx]

**PONE-D-22-18704**

**Examining doctors’ business analytics capabilities in using the electronic medical record system for decision-making effectiveness in intensive care units: impact of the COVID-19 pandemic**

**Authors’ responses to reviewers’ comments:**

Dear editors and reviewers,

First of all, thank you for providing us with the opportunity to submit a third revised version of our manuscript, *“Examining doctors’ business analytics capabilities in using the electronic medical record system for decision-making effectiveness in intensive care units: impact of the COVID-19 pandemic”,* for publication consideration in PLOS ONE.

We thank the 4^th^ reviewer for accepting our revision in the previous revised manuscript. We are very much encouraged by the kind and useful comments. In this version, our revision takes all the suggestions provided by the 4^th^ reviewer to improve our manuscript to a publication-ready version. Please find a summary of our revision on the following pages.

We are confident that you will find we have been able to address all of the reviewer’s comments satisfactorily and hope that you will find the third revised manuscript ready for publication in your esteemed journal. Thank you again for inviting us to re-submit our manuscript.

Best regards,

*Anonymous authors*

**Reviewers' comments:

Reviewer's Responses to Questions**

**Comments to the Author

1. If the authors have adequately addressed your comments raised in a previous round of review and you feel that this manuscript is now acceptable for publication, you may indicate that here to bypass the “Comments to the Author” section, enter your conflict of interest statement in the “Confidential to Editor” section, and submit your "Accept" recommendation.**

**Reviewer #4: All comments have been addressed**

**2. Is the manuscript technically sound, and do the data support the conclusions?

The manuscript must describe a technically sound piece of scientific research with data that supports the conclusions. Experiments must have been conducted rigorously, with appropriate controls, replication, and sample sizes. The conclusions must be drawn appropriately based on the data presented.**

**Reviewer #4: Yes**

**3. Has the statistical analysis been performed appropriately and rigorously?**

**Reviewer #4: I Don't Know**

**4. Have the authors made all data underlying the findings in their manuscript fully available?

The** [**PLOS Data policy**](http://track.editorialmanager.com/CL0/http:%2F%2Fwww.plosone.org%2Fstatic%2Fpolicies.action%23sharing/1/010f0192fe486a32-0f70669b-37a0-4578-9e6b-da4ff13f430f-000000/HJyOu8g89N4NPIQOn-GGMuu2liWq69lOVL4R6-Y0fNg=183) **requires authors to make all data underlying the findings described in their manuscript fully available without restriction, with rare exception (please refer to the Data Availability Statement in the manuscript PDF file). The data should be provided as part of the manuscript or its supporting information, or deposited to a public repository. For example, in addition to summary statistics, the data points behind means, medians and variance measures should be available. If there are restrictions on publicly sharing data—e.g. participant privacy or use of data from a third party—those must be specified.**

**Reviewer #4: Yes**

**5. Is the manuscript presented in an intelligible fashion and written in standard English?

PLOS ONE does not copyedit accepted manuscripts, so the language in submitted articles must be clear, correct, and unambiguous. Any typographical or grammatical errors should be corrected at revision, so please note any specific errors here.**

**Reviewer #4: Yes**

**6. Review Comments to the Author

Please use the space provided to explain your answers to the questions above. You may also include additional comments for the author, including concerns about dual publication, research ethics, or publication ethics. (Please upload your review as an attachment if it exceeds 20,000 characters)**

**Reviewer #4: Thank you for the opportunity to review this revised manuscript. I appreciate that the authors have addressed all of my comments in a comprehensive manner. I think the manuscript is easier to understand for those with less content expertise and more appropriate for a general medical audience.

Only a few minor grammatical suggestions below, but otherwise, I think the manuscript is much improved.

Introduction
Page 3 - "EMR system becomes a vital decision support tool" seem like an incomplete sentence after the revisions.

Also Page 3 - "More so in the ICU setting, where mortality and morbidity risks are higher than in other wards of a hospital." This is an incomplete sentence.

Methods
Page 16 - First para - "We design the questionnaire..." should be "We designed..."**

**7. PLOS authors have the option to publish the peer review history of their article (**[**what does this mean?**](https://track.editorialmanager.com/CL0/https:%2F%2Fjournals.plos.org%2Fplosone%2Fs%2Feditorial-and-peer-review-process%23loc-peer-review-history/1/010f0192fe486a32-0f70669b-37a0-4578-9e6b-da4ff13f430f-000000/1iSC4aeV7hE5fQRjsmwbhE4gT8LgQ3jyU7h8UESQhX4=183)**). If published, this will include your full peer review and any attached files.


If you choose “no”, your identity will remain anonymous but your review may still be made public.


Do you want your identity to be public for this peer review? For information about this choice, including consent withdrawal, please see our** [**Privacy Policy**](https://track.editorialmanager.com/CL0/https:%2F%2Fwww.plos.org%2Fprivacy-policy/1/010f0192fe486a32-0f70669b-37a0-4578-9e6b-da4ff13f430f-000000/r75TYOMBWqYzvIpK92Zny62YPJN8XzA-xnzJ5XHix3g=183)**.**

**Reviewer #4: No**

| **Reviewer 4 Feedback** |  |
| --- | --- |
| Thank you for the opportunity to review this revised manuscript. I appreciate that the authors have addressed all of my comments in a comprehensive manner. I think the manuscript is easier to understand for those with less content expertise and more appropriate for a general medical audience.  Only a few minor grammatical suggestions below, but otherwise, I think the manuscript is much improved. | Thank you for your comments. We very much appreciate your encouragement that the manuscript is now easier to understand for a general medical audience. Our revision considers all your suggested areas of improvement to improve our manuscript toward a publication-ready version.  We have copyedited our manuscript to enhance its structure and readability, addressing all the grammatical suggestions.  We also corrected the sequencing of our in-text citations to a correct order. You may find our amendments to this revised manuscript in our following responses to your comments.  Please be assured that while copyediting this third revised manuscript, we have also carefully preserved the improvements we made in our first revised manuscript. |
| Introduction Page 3 - "EMR system becomes a vital decision support tool" seem like an incomplete sentence after the revisions.  Also Page 3 - "More so in the ICU setting, where mortality and morbidity risks are higher than in other wards of a hospital." This is an incomplete sentence. | Thank you for your suggestion. We have revised the lines to  “In the care and management of critically ill patients, the EMR system becomes a vital support tool aiding doctors in treatment plans”.  “More so in the setting of the intensive care unit (ICU), where critically ill are cared for, decisions that affect mortality and morbidity outcomes are higher compared to the general wards which house relatively stable patients.” |
| Methods Page 16 - First para - "We design the questionnaire..." should be "We designed..." | Thank you for pointing out, we have corrected the grammatical error. |

--- End of Authors’ responses to reviewers’ comment ---
